# Correlation of TBE Incidence with Red Deer and Roe Deer Abundance in Slovenia

**DOI:** 10.1371/journal.pone.0066380

**Published:** 2013-06-11

**Authors:** Nataša Knap, Tatjana Avšič-Županc

**Affiliations:** Faculty of Medicine, Institute of Microbiology and Immunology, University of Ljubljana, Ljubljana, Slovenia; University of Texas Medical Branch, United States of America

## Abstract

Tick-borne encephalitis (TBE) is a virus infection which sometimes causes human disease. The TBE virus is found in ticks and certain vertebrate tick hosts in restricted endemic localities termed TBE foci. The formation of natural foci is a combination of several factors: the vectors, a suitable and numerous enough number of hosts and in a habitat with suitable vegetation and climate. The present study investigated the influence of deer on the incidence of tick-borne encephalitis. We were able to obtain data from deer culls. Using this data, the abundance of deer was estimated and temporal and spatial analysis was performed. The abundance of deer has increased in the past decades, as well as the incidence of tick-borne encephalitis. Temporal analysis confirmed a correlation between red deer abundance and tick-borne encephalitis occurrence. Additionally, spatial analysis established, that in areas with high incidence of tick-borne encephalitis red deer density is higher, compared to areas with no or few human cases of tick-borne encephalitis. However, such correlation could not be confirmed between roe deer density and the incidence of tick-borne encephalitis. This is presumably due to roe deer density being above a certain threshold so that availability of tick reproduction hosts has no apparent effect on ticks' host finding and consequently may not be possible to correlate with incidence of human TBE.

## Introduction

Tick-borne encephalitis (TBE), the most important viral tick transmitted disease in Europe and Russia is caused by tick-borne encephalitis virus (TBEV). In Slovenia around 300 cases are reported annually, with incidence around 14 per 100,000. Endemic area reaches over the whole northern part of the country, through the central part of the country to the south. In the last two decades, a number of new natural foci of TBE have appeared in Italy, Switzerland, Germany and other countries in Europe [1], [2], [3], [4], [5]. Additionally, in most countries in Europe, TBE incidence increased sharply from 1974 to 2003 [6]. The exception is Austria, where the number of cases decreased considerably due to an effective vaccination campaign [7], [8]. Furthermore, number of cases in Sweden increased dramatically and new foci were discovered further northwards in Finland, as well as at higher altitudes [9], [10], [11], [12]. The changes in incidence and spatial distribution occurred within a relatively short period of time throughout several countries in Europe, including Slovenia.

In the past decade several studies were published indicating important factors influencing the changes in TBE incidence. One of commonly mentioned factors was the climate change; increasing temperature raises winter survival rates and extends the developmental periods of ticks, specifically *Ixodes ricinus* [13], [14], [15]. Additionally it effects the duration of the vegetation period, thereby improving the suitability of the habitat for ticks and also affecting the tick hosts [11], [16]. Socio-economic changes and human behavior were the second set of factors indicated to influence the TBE incidence [17], [18], [19].

Small mammals play a central role in virus transmission between ticks and are important hosts in tick life cycle. As non-viremic host, they are essential for transmission of TBEV by co-feeding of infected and non-infected ticks [20], [21], [22], [23]. Additionally, viremia in small mammals is sufficiently high and most likely not as short termed as previously thought to enable direct transmission of the virus to feeding ticks [24], [25], [26], [27]. Deer on the other hand are not competent hosts for TBEV transmission, but they are important in the enzootic cycle of TBEV since they support tick populations [3], [28], [29], [30], [31]. They have often been neglected in studies of natural foci of TBEV. Recently, several studies have been conducted mainly on the importance of roe deer (*Capreolus capreolus*). Research shows that the increase in the abundance of roe deer leads to an increase in tick populations [32], [33], [34]. In fact, some new studies indicate, that change in deer population might be a pivotal factor influencing the *Ixodes ricinus* population, and henceforth the TBE virus [11], [31], [34].

The aim of our study was to determine the roles of two species of large hosts, red deer (*Cervus elaphus*) and roe deer (*Capreolus capreolus*), on the incidence of TBE. We wanted to examine whether the changing density of roe deer and red deer in time and space influences the incidence of TBE.

## Materials and Methods

We collected data on killed deer in Slovenia for the period from 1970 to 2008. Data were obtained from the Slovenian Hunting Association for the years 1970 to 2000. Data on killed deer after the year 2000 were obtained from the Slovenia Forest Service.

The data were used to estimate the size of deer populations in a given area. The number of animals predicted for the cull is determined on the basis of the analysis of past management and assessment of the state population (abundance, structure, interactions of populations of different species of deer and health status).

Data on TBE incidence in Slovenia for the period from 1970 to 2008 was collected from the Institute of Public Health of Slovenia and the Laboratory for diagnosis of Zoonoses at the Institute of Microbiology and Immunology in Slovenia.

Based on the information on the cull, we evaluated the size of the deer population and its density. The software package SPSS Statistics IBM 19 (© IBM Corporation, Somers, New York, USA) and R statistical software, version 2.15.1 were used for statistical analysis. Temporal link between TBE incidence and abundance of deer was determined using Pearson correlation coefficients. Time lags due to tick developmental cycle were considered, and therefore a correlation was considered between deer abundance and TBE incidence with a time lag (1–5 years). Additionally Ordinary least squares (OLS) regression was performed with TBE incidence and roe and red deer abundance (including interaction). Both the red deer and the roe deer abundance were first centred around their means (3117.9, 29545.9, respectively) to avoid collinearity between predictors, and their interaction was calculated. Neither the variance inflation factor nor the tolerance showed any collinearity between the centred predictors.

Additional spatial analysis was performed. Data on deer density and TBE incidence was available for Slovenia (204 units). Means of TBE incidence and red deer and roe deer density in the period 2005–2007 were calculated for these areas. Logistic regression was used to find out whether deer density discriminates between different groups TBE incidence.

## Results

**Figure 1:**
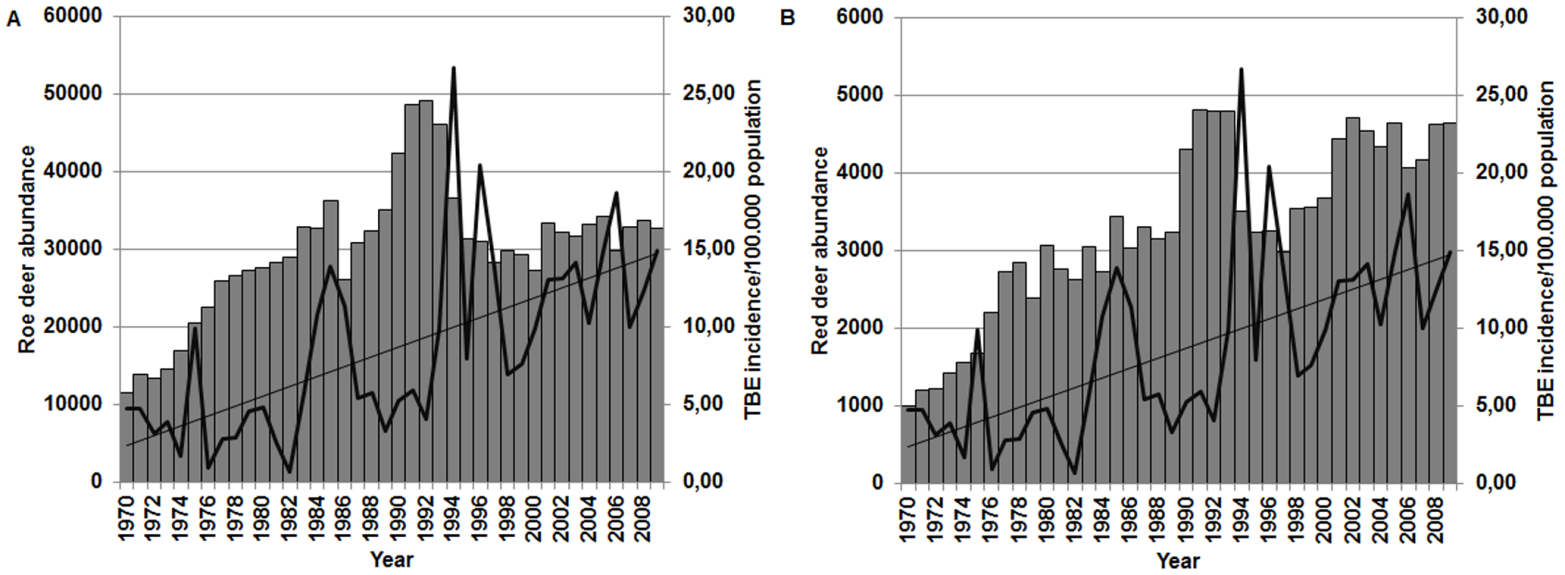
The abundance of deer compared with the incidence of TBE. A. The abundance of roe deer (columns) compared with the incidence of TBE (line) from 1970 to 2008. B. The abundance of red deer (columns) compared with the incidence of TBE (line) from 1970 to 2008.

In Slovenia TBE incidence shows an upward trend from 1970 on. A gradually rising trend was also observed in both, red deer and roe deer populations (Figure 1). For both roe deer and red deer, a correlation between deer abundance and the TBE incidence at different time lags was determined using Pearson correlation coefficient (Table 1). The highest positive linear relationship was found between the TBE at time t and red deer and roe deer abundance at time t-3 (r = 0.7, p<0.001 and r = 0.6, p<0.001., respectively). The regression model (OLS regression) with three predictors explained 47% of TBE incidence variance. Best predictor of the TBE incidence at time t is red deer abundance at time t-3. Roe deer abundance is not a significant predictor of TBE incidence; the red deer-roe deer abundance interaction shows borderline significance, suggesting that further analysis with larger sample size could clarify this aspect (Table 2).

**Table 1:** Correlation analysis using Pearson correlation coefficient.

Time (lag)	Red deer		Roe deer	
	R	p-value	R	p-value
t	0.4	0.012	0.2	0.153
t-1	0.6	<0.001	0.4	0.012
t-2	0.6	<0.001	0.5	0.003
**t-3**	**0.7**	**<0.001**	**0.6**	**<0.001**

Relationship between red deer/roe deer abundance and the TBE incidence at different time lags of TBE incidence. R – Pearson correlation coefficient; p-value (p-values <0.001 were considered significant).

**Table 2:** Regression analysis results – temporal analysis.

	Stnd. β	T	p-value
Constant	7.7		
**Red deer t-3, centred**	**0.005**	**3.1**	**0.004**
Roe deer t-3, centred	−0.0001	−0.6	0.57
Interaction	0.1^−6^	1.8	0.07
Adjusted R^2^	0.47		

Regression was performed using ordinary least squares (OLS) with TBE incidence at time t as the dependent variable and red deer and roe deer abundance (including interaction) at time t-3 as the predictor.The best predictor was red deer abundance and is shown here with standardized β coefficient (Stnd.β) the T- value (T) and p-value (p-values <0.05 were considered significant).

Spatial analysis was performed additionally. Hierarchical clustering with Euclidean distance as similarity measure and Ward's method was performed on TBE incidence to identify areas with similar TBE incidence. This procedure revealed two distinctive groups. Mean (range; median; standard deviation) TBE incidence in one of the groups was 7.3 (0–22.8; 5.9; 7.1) and 52.3 (25.5–149.5; 44.7; 27.7) in the other. Thus, low TBE incidence areas were joined in one group and higher TBE incidence areas in the other.

**Figure 2:**
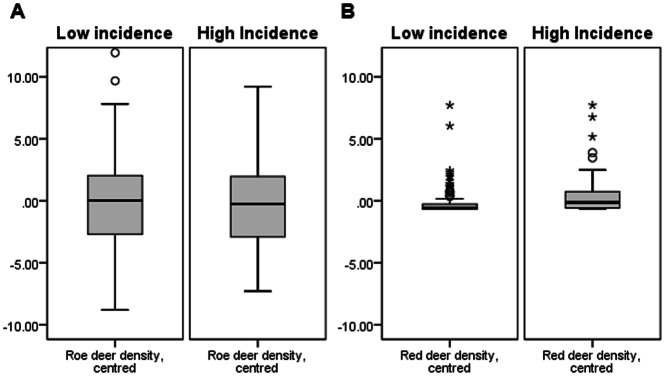
Deer density (centred) in areas with high and low TBE incidence. A. Roe deer density (centred); B. Red deer density (centred).

Logistic regression was used to find out whether the deer density discriminates between the two groups (Table 3). Roe deer and red deer density values were centred around their means (8.79; 0.67) and their product was calculated to avoid predictor multicollinearity. Odds ratios above 1 mean that higher values of predictor are associated with higher odds of higher TBE incidence. It can be seen that higher red deer densities are associated with increased odds of higher TBE incidence (Figure 2). Neither the roe deer density nor the red deer-roe deer density interaction contributed significantly to discrimination between the groups. These results are consistent with the results of the temporal analysis.

**Table 3:** Regression analysis results – spatial analysis.

Reference category: low TBE incidence group	OR (95% CI)	p-value
Red deer density (centred)	1.5 (1.11; 2.03)	**0.01**
Roe deer density (centred)	1.02 (0.94; 1.11)	0.60
Interaction effect	1 (0.91; 1.1)	0.98

Factors associated with higher TBE incidence, results of multivariate logistic regression. (OR – Odds ratio).

## Discussion

The importance of cervids (roe deer and red deer) in the circulation of TBE virus is mainly in their role as a host for ticks. Availability of suitable hosts is an important driver of tick abundance. Deer are often regarded as dilution host for transmission of the virus, because they are dead-end host. Ungulates are believed not to develop high enough viremia to enable transmission of the virus back to the feeding ticks [30], [35], [36], and the virus is considered to be non-pathogenic to deer [37]. The possibility of transmission of TBEV by co-feeding ticks on deer has been suggested because of the large number of ticks that can feed on deer at the same time in very close proximity [11], [37]. Nevertheless, there is no experimental or other direct evidence for non-systemic transmission of TBEV between ticks that feed on the deer.

Cervids play an important role in the life cycle of ticks, especially as one of the main hosts for adult female ticks [34], [38], [39]. Even though deer feed numerous ticks of immature stages, they are the most significant host for adult reproductive ticks [30], [34], [37], [38], [40]. If there are no deer, adults quest for the hosts with a much lower success rate and consequently the number of ticks eventually decreases. Accordingly, increasing number of deer has a significant effect on the expansion and abundance of ticks.

Previous studies that correlated deer densities with the abundance of tick data support the theory of the strong dependence between the abundance of ticks and deer numbers [3], [34], [37], [41]. On the other hand several published studies have not confirmed this connection [42], [43]. Some authors argue that the impact of these hosts on the abundance of ticks, mainly nymphs, is severely restricted when the number of deer exceeds a sufficiently low threshold. Due to the necessity of the second host, the influence of the large animal host population may be limited or blurred [44]. Nevertheless, a recently developed model shows that a significant increase in deer abundance leads to tick population increase, especially in areas where deer have been at low densities until that time [32]. Additionally, developed models indicate, that increasing the density of deer above specific threshold allows questing ticks to rapidly find a suitable host and thereby causing an apparent decrease in tick numbers [32]. Complete removal of deer may make it harder for the tick to complete its life cycle, since it does not have access to the large hosts. Experiments of deer exclusion have demonstrated the following: shortly after removal of deer from a sufficiently large area, more ticks can be found questing on vegetation as well as on small hosts, but over the next few seasons, there would be a decline in the number of ticks [30], [45]. Correspondingly, studies in Sweden, where tick abundance has been studied have found, that the increasing number of ticks can be explained by high availability of tick maintenance hosts, particularly roe deer, which has been increasing in the last three decades and the warmer climate, that influences the growing season, and consequently both the tick and deer [34]. Additionally, studies have confirmed a positive correlation between densities of roe deer and TBE infected ticks and TBE incidence [29], [31], [46], [47].

In our study we aimed to determine a correlation between deer density and the TBE incidence in Slovenia. Since a human TBE virus infection in Europe is usually due to the bite of a nymphal *Ixodes ricinus* tick, we compared the TBE incidence in a given year with the abundance of deer in the same year and a time lag of several years (one to five years) indicating a developmental cycle of the tick. The abundance of both deer species showed a correlation with TBE incidence over time. The incidence in year t and the abundance of deer three years prior (t-3) formed the best correlation indicating a time lag in the effect of deer population on TBE incidence (Table 1). Red deer exhibited a stronger effect on TBE incidence, especially in the developed regression model, where the species was shown to be a high and significant predictor (Table 2), whereas the predictive effect of both roe deer abundance and the additive effect of both species were not important.

A very detailed data set in the last decade enabled us to perform additional spatial analysis, where areas with high or low TBE incidence were linked with respect to deer density. Whilst the density of roe deer has not shown any link with the incidence of TBE, there is a significant correlation between red deer density and the incidence of TBE (Table 3). In areas with low density of red deer there were fewer reported human cases of TBE compared to the areas with high densities of deer (Figure 2).

Previous studies conducted in Europe have mainly concentrated on the link between roe deer and the incidence of TBE or the abundance of ticks [3], [28], [30], [31], [37], [48]. A study conducted in Italy demonstrated the presence of patients with TBE in the regions with higher density of roe deer. The study included only six provinces with known presence of TBE in humans and in nine provinces where no cases of human TBE have been reported. Average roe deer density in regions where TBE occurs was established at 4.34 heads per km^2^, whereas in areas with no human TBE cases was only 1.84 heads per km^2^ [31]. Our study was more extensive since it included the whole area of Slovenia, but we were unable to confirm this correlation. Possibly the significance of the impact of roe deer on the incidence of TBE in the Italian study increased due to the geographical limitations of the study or the absence of other suitable host species. In Slovenia we have a comparably low roe deer density only in a very limited area, possibly too restricted to detect a significant difference with the high density areas. It is possible, that the abundance of roe deer in Slovenia (average density is 8.79 heads per km^2^) exceeds the threshold beyond which the host density has no effect on the probability of host finding by ticks.

Red deer density in Italy has been reported to be from 0.28 to 0.75 heads per km^2^, which is a relatively low density. But red deer density was not demonstrated to be significant in that study [31]. In our study the density of red deer was much greater and showed considerable variation (from 0 to 8.39 heads per km2, with a mean of 0.67 heads per km^2^) which enabled us to show a significant effect of red deer on TBE incidence.

The abundance of red deer increased sharply in recent decades in Slovenia. Elsewhere in Europe, the abundance of this species is also increasing. In Slovenia, red deer were almost completely extinct in the mid-19th century, but in the early 20th century its numbers began to rise due to shooting restrictions and artificial restocking. Today, red deer are found throughout most of Slovenia, but with significant variations in density [49], [50].

Based on our results, TBE incidence is dependent upon on the density of red deer. Whether that is the consequence of some species specific characteristic that enables them to be better hosts for the tick or the virus itself still remains to be established. On the other hand red deer come into contact with ticks due to its life style more often than roe deer, since they prefer different habitats and consume different vegetation [51]. A third possibility is that the red deer coincidentally prefer areas which have for some other grounds enabled the maintenance of virus circulation (e.g., certain vegetation). It would therefore be reasonable to perform additional analysis to evaluate the influence of vegetation composition on the incidence of TBE.

Results of our study offer evidence, that not only roe deer, but also red deer should be considered as important reproductive as well as maintenance tick hosts. Previous studies supported the role of roe deer as the species responsible for influencing tick abundance, a consequence of their important role as the main host for the adult reproductive ticks [30], [34], [37], [38], [40]. Though our study did not offer conclusive evidence of a crucial role of roe deer on the incidence of TBE in Slovenia; this is most likely due to the fact that the species is so numerous in Slovenia, that it exceeds the threshold level above which there is no measurable effect on TBE incidence. Nevertheless the evidence supporting their role in the enzootic cycle of TBE is significant and they should therefore be regarded as an important factor contributing to the circulation of the virus in nature.

The enzootic cycle of TBE is very complex, and a lot of factors should be considered when trying to explain the changes which have appeared in the past decades in TBE distribution. Activity of humans is an important factor, as indicated in numerous studies [19], [52], [53]. Nevertheless, tick hosts and the climate are those factors which influence tick abundance and TBE virus transmission [9], [27], [54], [55]. This indicates that more attention should be given to the host species and their abundance in the areas where TBE virus is circulating. Therefore extensive studies are needed in order to establish unambiguous connections between large and medium host populations and TBE incidence.
